# Origin of mounds in the Pantanal wetlands: An integrated approach between geomorphology, pedogenesis, ecology and soil micromorphology

**DOI:** 10.1371/journal.pone.0179197

**Published:** 2017-07-12

**Authors:** Jairo Calderari de Oliveira Junior, Raphael Moreira Beirigo, Mariane Chiapini, Alexandre Ferreira do Nascimento, Eduardo Guimarães Couto, Pablo Vidal-Torrado

**Affiliations:** 1 Department of Agronomy, Federal University of Technology–Parana, Dois Vizinhos, Parana, Brazil; 2 Agricultural Science Center, Federal University of Paraíba, Areia, Paraíba, Brazil; 3 Department of Soil Science, University of São Paulo, Piracicaba, São Paulo, Brazil; 4 Embrapa Agrossilvipastoril, EMBRAPA, Sinop, Mato Grosso, Brazil; 5 Department of Soil and Rural Engineering, Federal University of Mato Grosso, Cuiabá, Mato Grosso, Brazil; University of California Santa Barbara, UNITED STATES

## Abstract

Vegetated mounds are an important geomorphological feature of the Pantanal, where the influence of floods dictates not only hydropedological processes, but also the distribution and ecology of the flora and fauna. This work aimed to identify factors and processes that influence the formation and spatial distribution of the mounds, which are commonly associated with termite activity. In order to characterize pedological processes, macro and micro morphological descriptions, satellite image interpretation, dating of the sandy sedimentary material using OSL and carbon dating using ^14^C AMS were carried out. This dating of the materials indicates that the sediments in which the soils were formed were deposited during the Pleistocene, while the carbonates are from the Holocene. The basin-like format of the laminar structures suggests that part of the more clayey material was deposited in lacustrine environments. The more humid climate in the Holocene intensified argilluviation, which at an advanced stage, led to a more pronounced textural gradient, reducing drainage and leading to ferrolysis and thickening of the E horizon. Besides pedogenic processes, more erosive flooding during the Holocene began reducing and rounding the landscape’s more elevated structures (paleolevees). In the final stage, these structures were occupied by termites to shelter from flooding. Thereafter, the bio-cementation action of the termite nests has increased the resistance of the vegetated mounds to processes of erosion.

## Introduction

The majority of studies on vegetated mounds in tropical environments recall their origin through biological activity [1–6]. Termites are regarded as being the most responsible for mobilization and homogenization of the solid phase of soil, resulting in horizons with diffuse transitions. Biological activity is also associated with increasing soil aggregate stability, due to incorporation of organic material and the formation of channels, which also increases soil drainage capacity [7–8].

Pfahler *et al*. [9] and Renard *et al*. [10], working with mounds on the coastal plain of Guyana, suggest that their origin is mixed, initially being built by humans for food production in the pre-Colombian era and later being colonized by social insects. Even after the abandonment of these areas by man, the mounds persisted on the landscape as a result of biological activity, with the insects structuring and strengthening the soil and, consequently, increasing their resistance to erosive processes.

Silva *et al*. [11], utilizing carbon isotopic signature (δ^13^C), observed that the material present in the termite nests had a predominance of *cerrado* vegetation, refuting the hypothesis that the termites had built the mounds up on floodplains in which field vegetation predominates (C4 type species). Mathews [12] suggests that the mounds of Brazilian savannas susceptible to flooding result from a succession of different species of termites. At first, species more tolerant to humid environments built up nests, creating a higher drier environment. With the demise of the colony, the nest was then available to be colonized by another more flood sensitive species, which further increased the mound size.

However, the role of hydropedological processes in mound formation has been neglected by the majority of these studies, resulting in the historical dissemination of termites as being mostly responsible for the process [13]. Nevertheless, a hybrid concept of mound formation, combining biotic and pedogenic factors, has become more robust in its consideration of multiple and successive processes [5], and certain soils and geomorphological attributes may play an important role.

Soil analyses can provide essential information for better understanding of mound genesis. Morphology shows the environmental conditions and pedogenic processes that soil has undergone, such as waterlogging, clay translocation, biological activity, etc [14]. Undisturbed samples are useful to understand which pedological process was produced at which past time (preterite) or is occurring nowadays. The arrangement of soil components (matrix, pores, structures, etc.) also affects water movement. Both pedological processes and water movement can be interpreted through morphological description of thin sections at microscopic scale.

In depositional environments, soil particle-size analyses may provide information on soil texture differences with depth, whereby insignificant differences suggest that soil horizons were formed by pedological processes [1]. Wide variations in soil texture may have resulted from sediment deposition under different flood conditions, of higher or lower energy. Chemical attributes may also provide evidence of soil behavior or processes to which it may have been subjected. High pH values could suggest low ion lixiviation or even ion concentration in soil, while high exchangeable soil percentage (ESP) promotes colloid dispersion, making the soil more susceptible to erosion.

Nevertheless, according to Ponce and Cunha [15], in mounds (*capões* or *murundu*) differences can be observed between termite caste material and soil without biological activity. Moreover, the volume of soil in mounds is often superior to that contributed and used by termites in their activities [16]. Studying the genesis of the mounds randomly distributed in the Northern Pantanal, Nascimento *et al*. [1] suggest that some features observed in the profile and on thin sections, illustrate the importance of termite activity in this process. However, little attention has been given to soil attributes resulting from pedogenic processes, which, in turn, may also lead to the creation of mounds.

The objective of this work is to identify the influence of other factors beyond the biological, such as hydropedological, in the continuing evolution of mounds on Pantanal wetlands, particularly those occurring in aligned rows on the floodplain. The configuration of aligned mounds is likely associated with erosive processes linked to the geomorphological and pedological evolution of the fluvial plain.

## Material and method

### The study area

**Fig 1:**
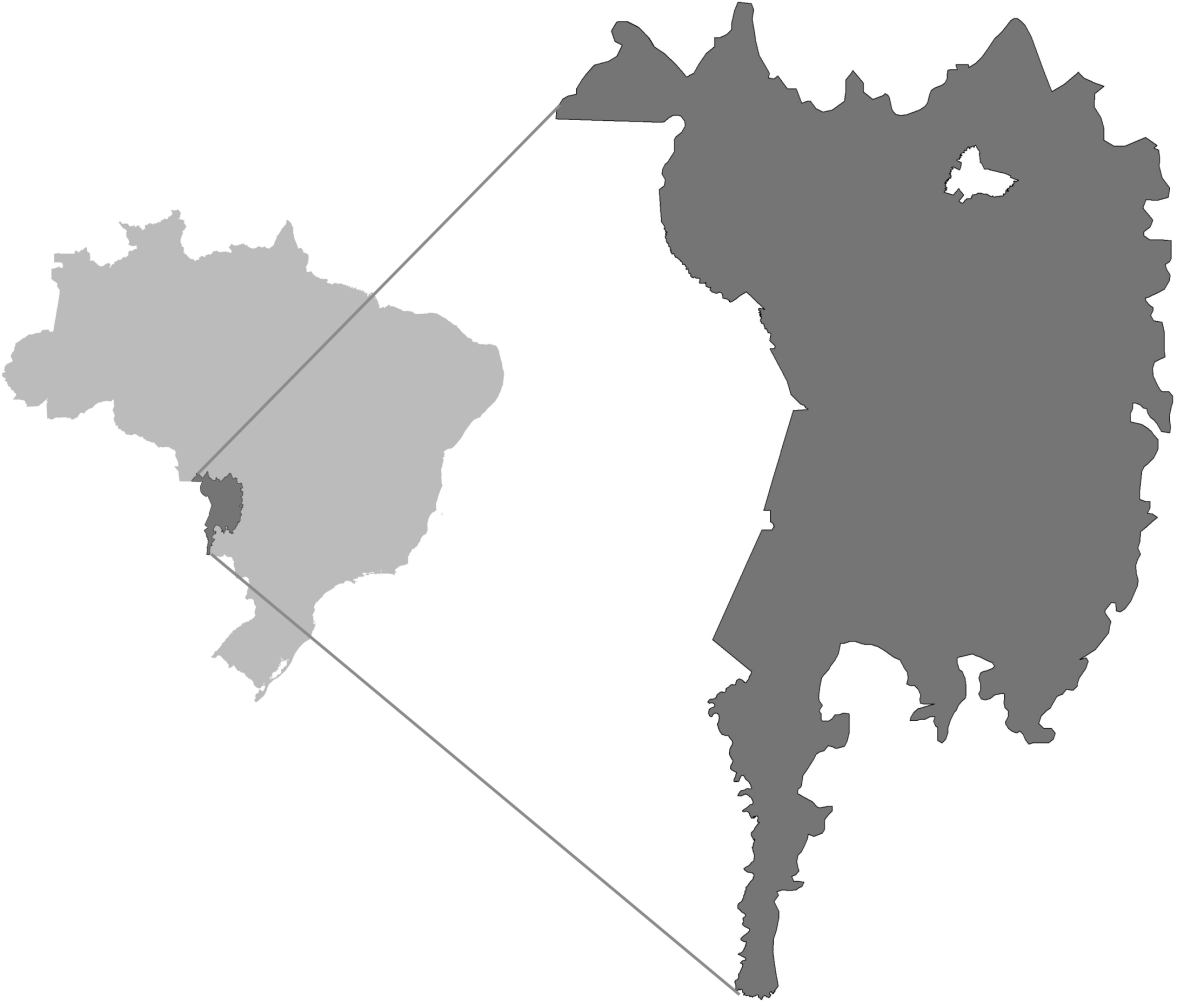
The Brazilian Pantanal (light grey) and the study area (white area) in Northern Pantanal (dark grey).

The study was conducted in the Private Natural Heritage Reserve [*Reserva Particular do Patrimônio Natural*] (RPPN) belonging to the Pantanal Social Service of Commerce [*Serviço Social do Comércio*] (SESC), in the municipality of Barão de Melgaço, North Pantanal. The area is located between the coordinates of 16°32’S to 16°49’S and 56°03’W to 56°26’W (Fig 1). We would like to thank SESC Pantanal for the permission and essential logistic support to do this research.

**Fig 2:**
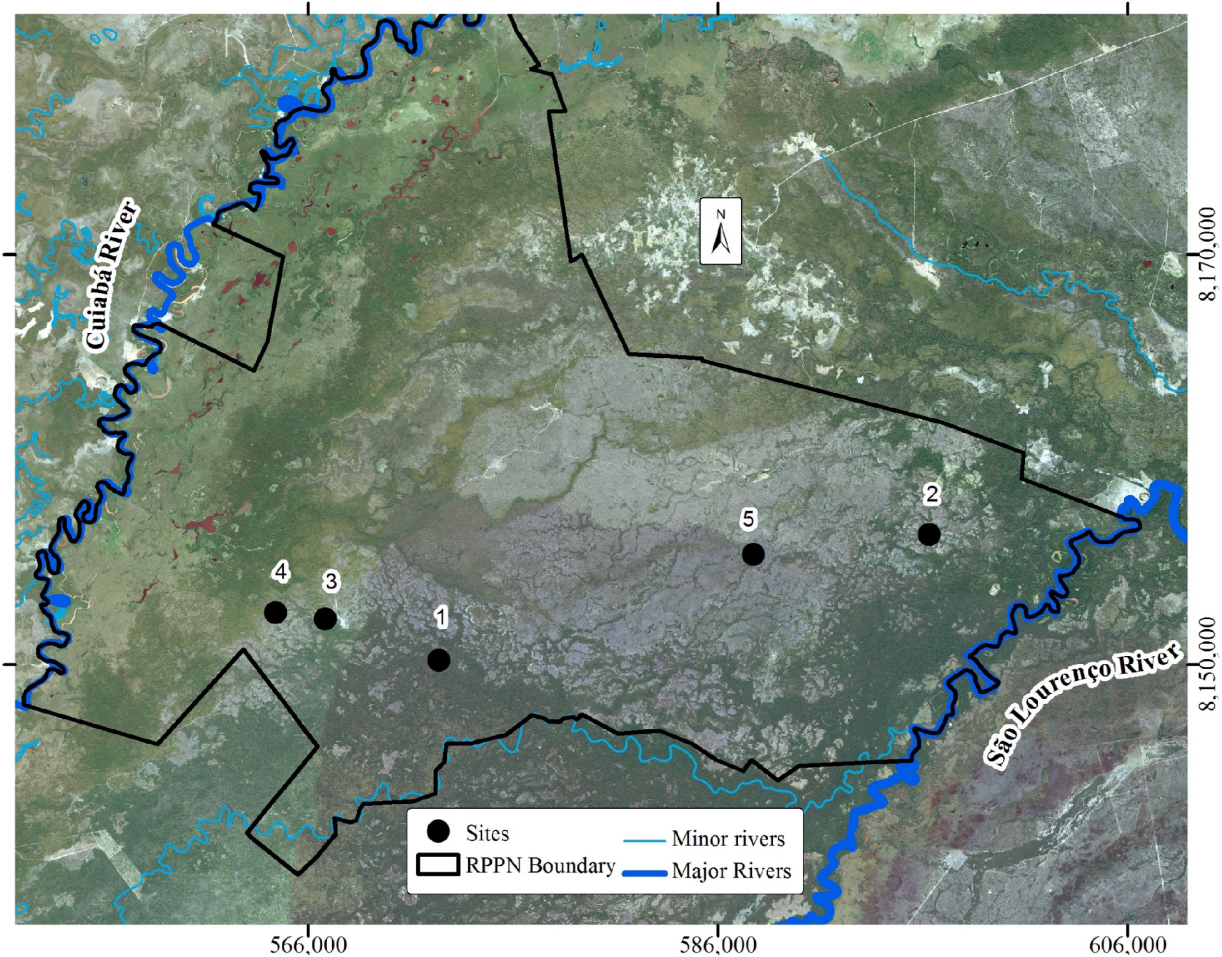
Study area and its boundary. Study area bordered to the west by the river Cuiabá and to the east by the São Lourenço river, study sites are indicated by stars (1 to 5). Coordinates in UTM projection system 21K zone (south). Printed under a CC BY license, with permission from “Secretaria de Estado de Planejamento do Mato Grosso” (SEPLAN-MT), original copyright 2008.

The RRPN is delimited to the west by the Cuiabá River and to the east by the São Lourenço River, totaling a little over 140,000 hectares (Fig 2). The climate of the region is tropical, classified as Aw according to the Köppen classification, with dry winters and rainy summers; the average annual rainfall is 1,500mm and evapotranspiration is 1,600mm, with the average monthly temperature varying from 22 to 32°C [17].

A wide variety of phytophysiognomies can be observed in the RPPN, from species characteristic of the semi-arid climate to vegetation types associated with flood environments, with a predominance of savanna vegetation identified by Hasenack [17] as follows: *Cerrado strictu sensu* (Savanna formations), *Cerradão* (Savanna forest formations), *Cambarazal forest* (*Vochysia divergens*), Seasonal Forest with *Acuri* (*Attalea phalerata*) palm trees, Fields and ecotones.

The *Pantanal* of Mato Grosso (Brazil), which extends beyond the borders of Bolivia and Paraguay, is the largest continental sedimentary floodplain in the world. The annual flooding of Pantanal plains occurs from November to April and the remaining months are marked by severe drought [17]. This phenomenon is a result of the low local inclination and the large volume of direct precipitation in the area and high water input from the rivers which drain the plateaus of the surrounding region. However, precipitation distribution is not uniform during flooding, but in the form of flood “pulses” and ebbs [18–20].

This alternation between flooding and drought dictates the balance among the diverse system components [21], resulting in a mosaic of ecosystems with a predominance of savannas and the occurrence of forests [17]. The sediments responsible for the geomorphological architecture of the Pantanal are directly affected by erosive, depositional and transportation processes [22–23], as well as by the dissolution and precipitation of chemical and mineral compounds [24].

Rivers which have migrated their channel, leaving their former channel abandoned (oxbow), dominate a large part of the Pantanal [25]. The principal formations they have left behind are i) floodplains–lower part of the landscape, with a predominance of grasses, which are the first to receive water during flooding season; ii) *Capões* (Savanna) and *Cordilheiras* (savanna ridges)–former paleolevee, which are the highest elevations, around 2 to 6 meters higher than the surrounding land and oval in shape with vegetation typical of the *cerrado* (savanna); their width and length varies from tens to hundreds of meters and they are rarely affected by flood waters; iii) *Murundu* (vegetated mounds)–areas punctuated by small vegetated elevations of approximately 1 meter with a predominance of dense *cerrado* vegetation, occasionally affected by flooding [15, 26–27].

### Study site selection

**Fig 3:**
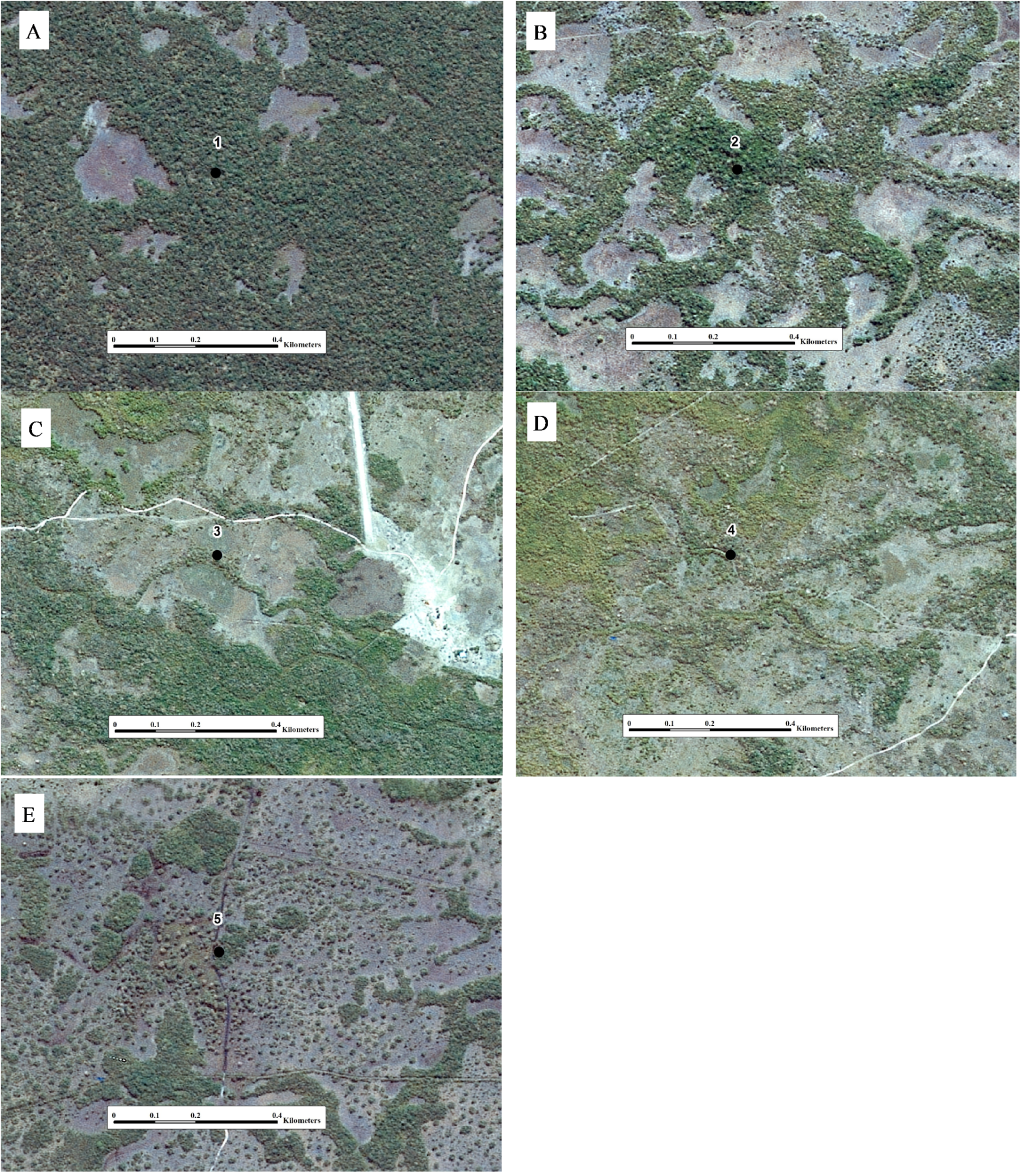
Satellite images from studied sites showing their geomorphological context. Close on site 1 (A), 2 (B), 3 (C), 4 (D) and 5 (E). Printed under a CC BY license, with permission from “Secretaria de Estado de Planejamento do Mato Grosso” (SEPLAN-MT), original copyright 2008.

In order to explore the relationship between landscape evolution and changes in soil attributes, study sites were selected through SPOT satellite image interpretation (Fig 2), with spatial resolution of 2.5 m and image composition made with bands 3, 2 and 1 (R3G2B1). Floodplains were identified as lighter in color with smooth image texture, while the greener color and rougher texture correspond to higher parts of the landscape associated with mounds. Mound size and distribution were used to identify different erosion stages, the smallest mounds being considered as more eroded. Five sites at different erosion stages were chosen for the study—P1, P2, P3, P4 and P5 (Fig 3). The satellite images were provided by “Secretaria de Estado de Planejamento do Mato Grosso” (SEPLAN-MT).

P1 is the most preserved mound. Lateral drainage occurs very slowly due to the “barriers” built by large mounds (Fig 3A). The floodplain remains isolated and waterlogged for long periods. There is a gradual transition between mound and floodplain.

P2 is situated in the north-east of the RPPN, this site is at a more advanced erosion stage than the previous. Floodplains are larger than site 1, but remain isolated by large mounds and lateral drainage is restricted (Fig 3B).

P3 presents narrower more elongated mounds (Fig 3C). Floodplains are larger and more connected, favoring lateral drainage. Mound-floodplain transition is abrupt, horizontal distance between the highest part of the mound and the waterlogged surface is around 6m and vertical distance is about 2m. To the south of the studied mound, there is a structure like a closed floodplain, resembling that observed at site 1, at an incipient stage of erosion.

P4 is the study site farthest from the São Lourenço river and closest to the Cuiabá floodplain, presenting larger narrow mounds and more connected floodplains (Fig 3D).

**Fig 4:**
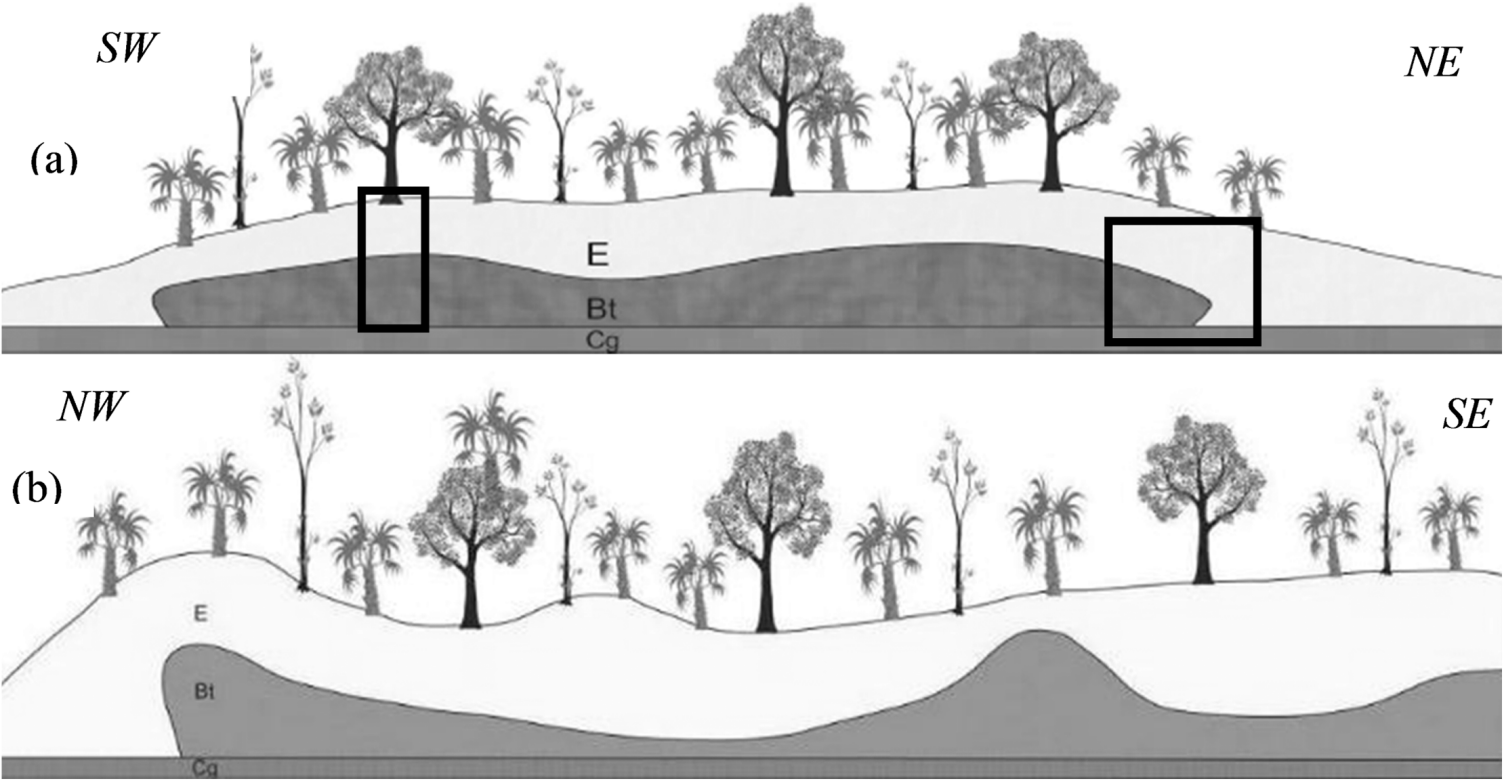
Schematic drawing of mound surface and top Btgn topography at site 5. Btgn topography was identified by augering, (A) in an orthogonal and (B) longitudinal view. The black rectangles indicate the location of the 5A (left) and 5B (right) trenches.

P5 is at a more advanced stage of erosion; mounds do not hamper lateral drainage and most of them are aligned. Floodplains make up the largest part of the surface, resulting in free drainage (Fig 3E). Flooding occurs in shorter periods than previous sites and water is lost mainly by lateral drainage and evapotranspiration is reduced. Mound-floodplain transition is sharp and two trenches were opened, one on the highest part of the mound, and another at the mound-floodplain transition (Fig 4).

### Field study and sampling

At each site a trench was opened on the mound (paleolevee) and morphological description of the soil profile on the mound was conducted according to FAO [28]. Samples were collected from each soil horizon (a total of 24 samples), air-dried and sieved through 2-mm-mesh sieves for chemical and particle-size analyses (soil texture).

Based on morphological description, horizons with increasing clay content or carbonate precipitation were chosen for undisturbed sampling and subsequent thin section confection. Once completely dry, undisturbed blocks were impregnated with a solution containing a mixture of polyester resin (Arazyn 1.0 –Ashland) and Styrene monomer at a proportion of 1:1, with added fluorescent pigment Tinopal OB (BASF®) and catalyst (Butanox) in a vacuum system, according to Murphy [29]. After hardening of the resin, the block was cut into sections with dimensions of 18 x 70 x 50 mm, stuck on a glass slide and polished to a thickness of 20–30μm for observation under a petrographic microscope. Major features, such as argilluviaton, carbonate nodules, iron/clay depletion or concentration, pore walls and connectivity, are essential to understanding the soil changes as erosion becomes more intensive (through P1 to P5). When observed on thin sections, these features were photographed in planar, polarized and UV-light. Soil micromorphology was described according to Bullock *et al*. [30] and Stoops [31].

### Chemical and particle-size analyses

The hydrometer method was used for particle-size analysis. The pH was determined in water (potentiometer), using a soil:solution ratio of 1:2.5 after agitation and 1-hour rest. Exchangeable aluminum (Al^3+^), potential acidity (H+Al), and exchangeable cations (Ca^2+^, Mg^2+^, K^+^, and Na^+^) were determined as described by Embrapa [32]. The Ca^2+^, Mg^2+^ and Al^3+^ were extracted with 1 mol l^−1^ KCl; K^+^ and Na^+^ with 0.0125 M H_2_SO_4_ + 0.05 M HCl; H+Al with 0.5 M calcium acetate at pH 7.0. Concentrations of Ca^2+^ and Mg^2+^ were determined by atomic absorption spectroscopy; K^+^ and Na^+^ by flame photometry; Al^3+^ and H+Al by complexometric titration. The results of the chemical analyses enabled calculation of the cation exchange capacity [CEC = Ca^2+^ + Mg^2+^ + K^+^ + Na^+^ + (H + Al)], the sum of exchangeable bases (S = Ca^2+^ + Mg^2+^ + K^+^ + Na^+^), base saturation [V = (Ca^2+^ + Mg^2+^ + K^+^ + Na^+^) × 100 / CEC], aluminum saturation [m = Al^3+^ × 100 / (Ca^2+^ + Mg^2+^ + K^+^ + Na^+^ + Al^3+^)], and exchangeable sodium saturation (ESP = Na^+^ × 100 / CEC) [32].

### Dating of the sediments and nodules of CaCO_3_

In order to see if the studied soils had recently undergone climatic change, during the Pleistocene or Holocene (from 2.58 Ma until today), samples of sandy layers were collected from Sites 2, 3 and 5 in PVC tubes and these were dated using optically stimulated luminescence (OSL), following the single-aliquot–regenerative dose (SAR) protocol proposed by Walinga *et al*. [33], at the Laboratory of Dating, Commerce and Rendering of Services Ltd [Laboratório de Datação, Comercio e Prestação de Serviços LTDA] in São Paulo. The nodules of calcium carbonate from Site 2 were sent to the Beta Analytic Inc laboratory for C^14^ dating using Accelerator Mass Spectrometry (AMS). It was not possible to collect samples for dating from the other sites, as these did not have a sandy layer at more than 50cm deep or precipitations of carbonate.

## Results

### Morphology and soil attributes

**Fig 5:**
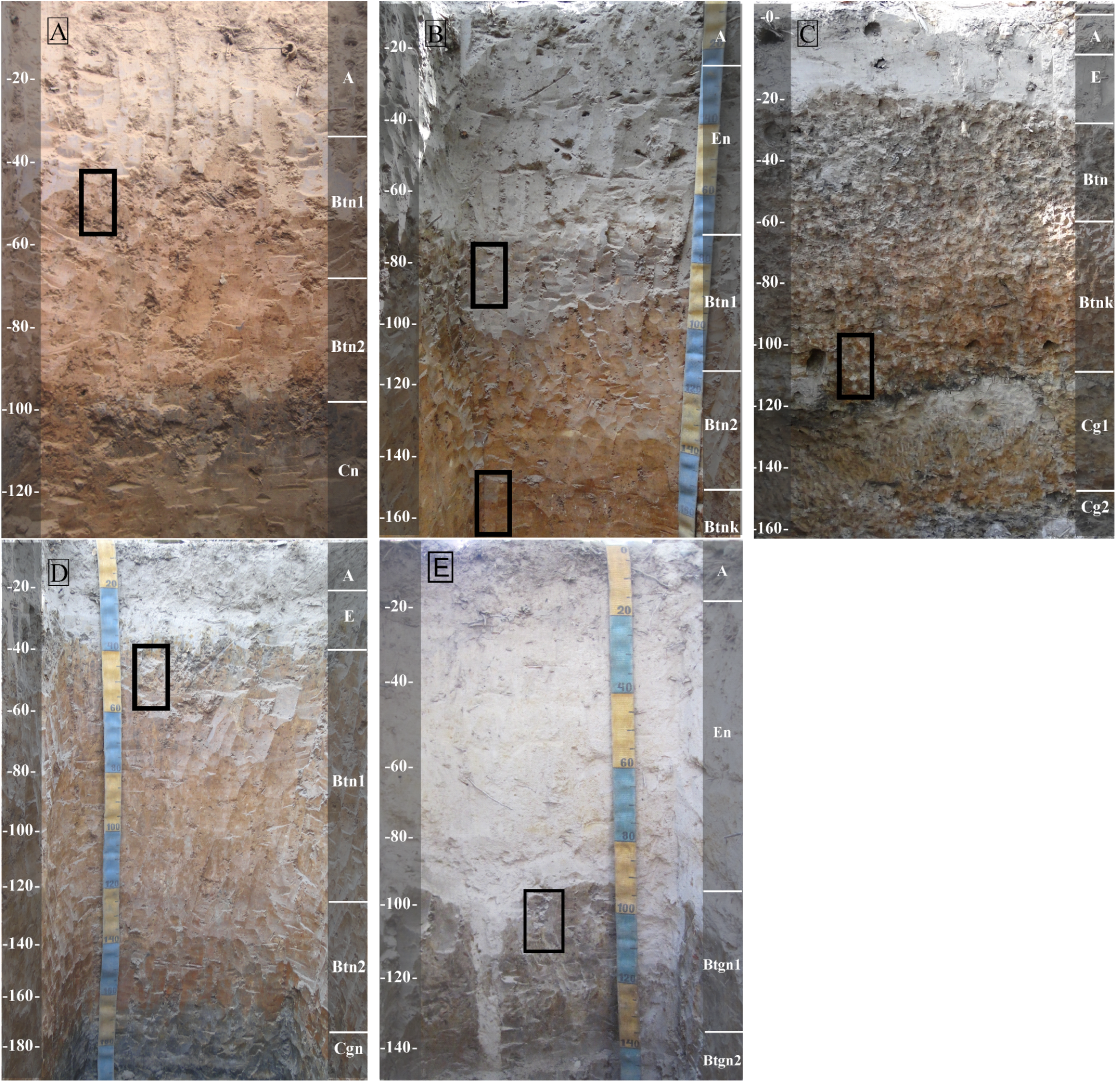
Soil profiles corresponding to five study sites. Profiles from study sites 1 (A), 2 (B), 3 (C), 4 (D) and 5 (E). Undisturbed sample sites for the thin sections are indicated by black rectangles.

In all soil profiles from the mounds we found a natric horizon, with Na^+^ and clay accumulation (illuviation) in the subsurface (Fig 5, Table 1). As the mound becomes more eroded, the color in the natric horizon is paler (Table 2), pH values tend to decrease and ESP increases. With the exception of site 1, all the soil profiles present a horizon depleted in clay and organic matter, while the sandy fraction was enriched (E horizon). This horizon stands out in site 5, where it is thickest and growing inside the natric horizon (Bt). Calcium carbonate was observed in old channels or fissures with a powdery consistency, only at sites 2 and 3.

**Table 1:** Soil classification and most important attributes for each site.

Site	IUSS Work Group WRB (2015)	Horizon	Depth^1^	Clay^2^	Silt^2^	Sand^2^	pH^3^	EC^4^	CEC^5^	ESP^6^
**1**	**Abruptic Solonetz** (Loamic, Cutanic, Magnesic, Hypernatric)	A	35	15	27	58	6.2	0.74	03.9	03
		Btn1	70	32	38	30	8.8	1.58	11.2	20
		Btn2	105	23	02	75	8.8	1.79	13.5	22
		Cn	>200	18	44	38	9.1	0.95	05.3	28
**2**	**Abruptic Solonetz** (Loamic, Cutanic, Magnesic, Hypernatric)	A	25	10	29	61	6.6	0.41	12.7	00
		En	65	10	29	61	8.0	0.04	03.7	26
		Btn1	100	30	67	33	8.9	0.76	09.7	22
		Btn2	150	28	42	30	9.5	0.87	08.7	32
		Btnk	>200	30	57	13	9.7	1.15	14.5	38
**3**	**Abruptic Stagnic Solonetz** (Albic, Loamic, Cutanic, Magnesic, Hypernatric)	A	10	09	24	67	6.5	0.46	06.8	08
		E	25	07	07	86	6.7	0.17	03.5	09
		Btn	60	26	23	41	9.1	0.49	12.9	34
		Btnk	115	17	22	61	8.8	0.20	12.9	35
		Cgn1	130	09	06	85	7.9	0.11	04.5	35
		Cgn2	180	16	01	82	6.7	0.07	05.7	33
**4**	**Abruptic Stagnic Solonetz** (Albic, Loamic, Cutanic, Magnesic, Hypernatric)	A	15	05	17	78	6.2	0.34	00.9	02
		E	35	05	21	74	7.1	0.05	00.4	05
		Btn1	120	30	52	22	8.9	0.71	10.8	60
		Btn2	155	35	58	07	8.7	0.60	15.4	51
		Cgn	>200	45	25	30	8.2	0.63	12.7	59
**5-A**	**Abruptic Stagnic Solonetz** (Albic, Loamic over Clayic, Cutanic, Magnesic, Hypernatric)	A	20	18	02	80	5.3	0.21	00.6	00
		En	100	15	05	80	6.6	0.12	02.0	33
		Btgn1	130	38	08	54	6.3	0.45	07.2	49
		Btgn2	160	47	09	44	6.0	0.23	05.0	51

^1^Bottom horizon limit in cm

^2^Total Clay and Sand content as a %

^3^pH measured in 1:2.5 soil/water ratio

^4^Electrical conductivity in saturated soil-paste (mS dm^-1^)

^5^Cation Exchange Capacity (cmol_c_ kg^-1^ of soil)

^6^Exchangeable Sodium Percentage, calculated on CEC.

**Table 2:** Soil classification and morphological attributes for each site.

Site	IUSS Work Group WRB (2015)	Horizon	Depth^1^	Distinctness^2^	Topography^3^	Matiz^4^	Hue/Chroma^4^	Nodules^5^	Thin^6^	Dating^7^	Age^8^
**1**	**Abruptic Solonetz**	A	35	Ab	Sm	10YR	6/4	-	-	-	-
		Btn1	70	Gr	Sm	7.5YR	4/6	-	X	-	-
		Btn2	105	Ab	Sm	7.5YR	6/6	-	-	-	-
		Cn	>200	-	-	7.5YR	4/3	-	-	-	-
**2**	**Abruptic Solonetz**	A	25	Gr	Sm	10YR	4/2	-	-	-	-
		En	65	Ab	Sm	7.5YR	6/3	-	-	-	-
		Btn1	100	Gr	Sm	7.5YR	4/6	-	X	-	-
		Btn2	150	Gr	Sm	7.5YR	5/8	-	-	-	-
		Btnk	>200	-	-	7.5YR	6/8	k	X	AMS/OSL	660/52,400
**3**	**Abruptic Stagnic Solonetz**	A	10	Gr	Sm	7.5YR	7/1	-	-	-	-
		E	25	Ab	Sm	10YR	8/1	-	-	-	-
		Btn	60	Gr	Ir	7.5YR	5/3	-	-	-	-
		Btnk	115	Gr	Ir	7.5YR	5/6	k	X	-	-
		Cgn1	130	Ab	Ir	7.5YR	6/2	-	-	-	-
		Cgn2	180	-	-	7.5YR	6/2	-	X	OSL	21,500
**4**	**Abruptic Stagnic Solonetz**	A	15	Gr	Sm	10YR	6/4	-	-	-	-
		E	35	Ab	Sm	10YR	8/1	-	-	-	-
		Btn1	120	Gr	Sm	7.5YR	4/6	-	X	-	-
		Btn2	155	Gr	Sm	7.5YR	5/6	-	-	-	-
		Cgn	>200	-	-	7.5YR	4/2	-	-	-	-
**5-A**	**Abruptic Stagnic Solonetz**	A	20	Gr	Sm	7.5YR	7/1	-	-	-	-
		En	100	Ab	Br	10YR	8/1	Mn	X	-	-
		Btgn1	130	Gr	Ir	7.5YR	4/1	Mn	X	-	-
		Btgn2	160	-	-	10YR	4/8	-	-	OSL	40,000

^1^Bottom horizon limit in cm

^2^Distinctness: Ab = Abrupt, Gr = Gradual

^3^Topography: Sm = Smooth, Ir = Irregular, Br = Broken

^4^Color notation in Munsell Soil Chart Color

^5^Nodules: k = carbonate, Mn = Manganese and Iron

^6^Thin section–horizons from which undisturbed sample was collected

^7^Dating–corresponding horizon from which sandy material was collected for OSL dating or carbonate material was collected for AMS dating.

^8^Age in years measured by AMS/OSL method.

**Fig 6:**
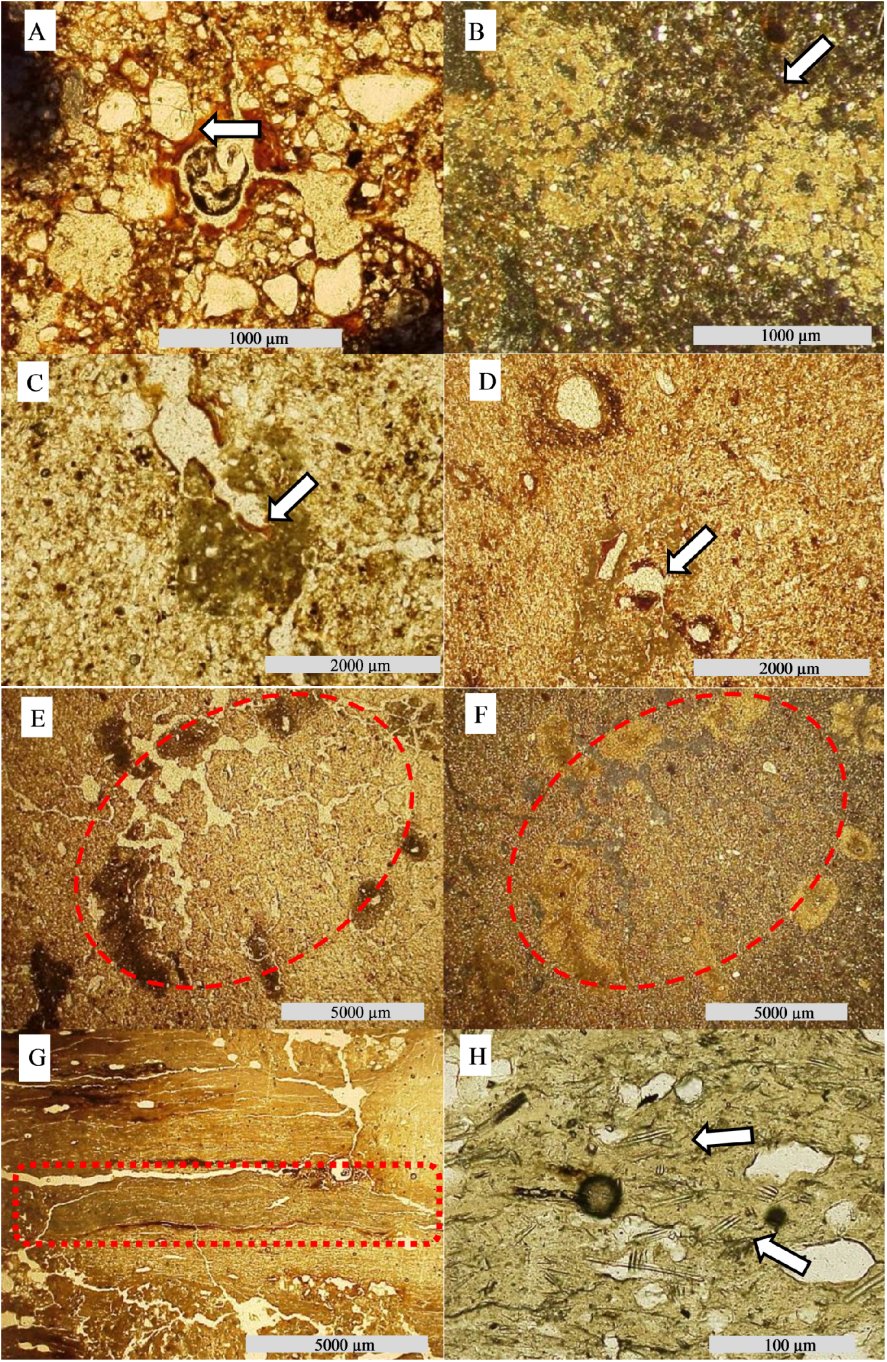
Photomicrographs from soil thin sections. (A) Btn1 horizon—iron and clay hypocoating (arrows—PPL). (B) Btnk horizon from Site 2 –calcite nodules cluster (XPL). (C) Fe coating on calcite nodules (PPL). (D) Fe infilling on calcite nodules (PPL—white arrow). (E) Nodules of calcite at different stages of degradation, in circular form (PPL), (F) the same feature in XPL. (G) Laminar structure of carbonate (red rectangle) (PPL). (H) Exoskeletons of diatoms (white arrows) imbedded in carbonate.

**Fig 7:**
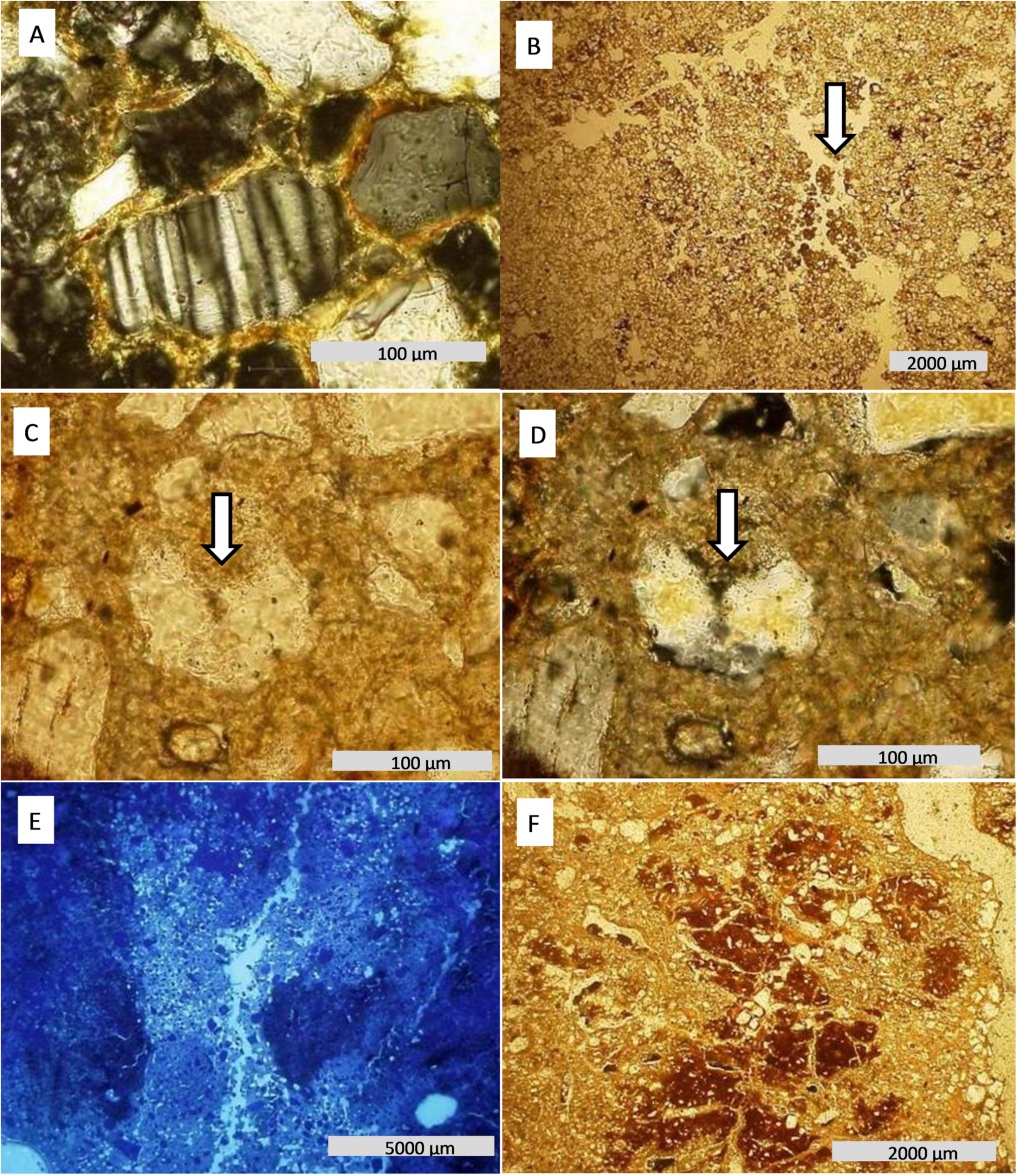
Photomicrographs from soil thin sections. (A) Btnk from site 3: weathering of plagioclase. (B) Calcite precipitation fragmented in Btnk horizon, associated with channels. (C) Dissolution of quartz grains amid carbonate matrix (PPL), (D) the same features in XPL. (E) Removal of fine material associated with pedal porosity. (F) Fe depletion related pedal pores, suggesting epissaturation process.

Features formed through pedogenic processes (pedofeatures), such as pore and channel walls coated with Fe or clay material (illuviation), Fe impregnation of the matrix inside pore walls (hypocoatings) and Fe and Mn removal from the soil matrix (depletion) were commonly observed on thin sections (Table 3). Carbonate remobilization through chemical dissolution is evident at sites 2 and 3 (Figs 6 and 7).

**Table 3:** Micromorphological description from thin sections, according to Bullock *et al*. [30] and Stoops [31].

Sample	Site 1 –Btn1	Site 2 –Btn1	Site 2 –Btnk	Site 2 –C	Site 3 –Btnk	Site 4 –Btn1	Site– 5 En/Btgn	Site 5 –En/Btgn
							Zone 1—Btgn	Zone 2 –En
**Microstructure**	Chamber	Chamber	Subangular blocky	Massive	Subangular blocky	Massive	Massive	Spongy
**Groundmass**								
**coarse material**	Quartz (100%)	Quartz (100%)	Quartz (100%)	Quartz (100%)	Quartz (95%)	Quartz (100%)	Quartz (100%)	Quartz (100%)
					Plagioclase (2%)			
					Feldspar (2%)			
					Biotite (1%)			
**Micromass**	Undifferentiated	-	Speckled	Speckled	Speckled	Speckled	Undifferentiated	Undifferentiated
				Poroestriated				
**c/f related distribution**	Chitonic	Single spaced porphyric	Porphyric	Single spaced porphyric	Gefuric	Close porphyric	Close porphyric	Concave gefuric
**Pedofeatures**	Clay coating	Fe hypocoating	Fe hypocoating	Fe/Mn hypocoating	Fe hypocoating	Clay coating	Fe hypocoating	Fe coatings
	Clay infilling	Clay infilling	Carbonate nodules	Fe/Mn quasicoating	Carbonate infilling		Fe-clay infilling	Mn coatings
	Fe hypocoating			Carbonate lens				

The most intensive Fe depletion and clay illuviation were observed at site 5. The related distribution between coarse and fine particles was identified as predominantly porphyric, with few voids or connected channels porphyric, while horizons with more porosity were identified as having a chitonic distribution. Sandy material was observed in the entire profile at the edge of site 5, coinciding with the lateral edges of the Bt horizon. No significant termite activity was observed at the five studied sites, neither through field observation nor micromorphological features such as excrement in voids or channels. Specific descriptions of the 5 sites are given below:

#### Site 1

Soil morphology presents clear smooth transition for all horizons (Fig 5). Subsuperficial Btn1 and Btn2-horizons have approximately twice as much clay content as the superficial A-horizon, associated with high pH values (8.8 for both) and high ESP (20 and 22%, respectively) (Table 1), identifying Bt as natric [28], and the soil profile as Abruptic Solonetz [34].

Through visual examination in the field, we observed a depression in the central part of the mound, aligned to its extension. Near the depression wildlife was found to practice intentional ingestion of soil, known as geophagy (*geo*–earth, *phagy*–feeding on), this therefore configures a geophagic site. The main reasons for wildlife soil consumption are mineral nutrition (mainly sodium) or dietary detoxification (toxin adsorption by clay minerals) [35]. Once arboreal species had fallen down, roots were exposed, as was the natric clayey horizon, enabling the geophagy [36]. Geophagy is common at several sites in the RPPN, although it is always associated with mounds, with high Na^+^ and clay content.

For the thin section, an undisturbed sample was collected from Btn1. In the micromorphological description (Table 3), the coarse/fine (c/f) related distribution pattern was observed as chitonic, with clay film binding coarse material [30]. Fe concentration was observed just below pore and channel walls (hypocoating) and was covered by a posterior clay accumulation in wall structures (clay coating). The clay coating is mainly composed of impure reddish clay (Fig 6A), which is non-laminated, associated with voids where well oriented clay results in a sharp extinction of polarized light and clear boundaries [30]. Coarse material is composed exclusively of quartz with little weathering.

#### Site 2

Geophagy was also observed at this site. The described profile presents a 40cm thick E horizon, which is followed in depth by Btn1- and Btn2-horizons until 150cm (Table 1). Underlying Btn2, a Btnk horizon has the same color and clay content (Table 2), with dispersed powdered lime (calcium carbonate) associated with fissures, which was observed with effervescence when put in contact with HCl [28]. All horizons, except the superficial, show high pH values (alkaline), ranging from 8.0 to 9.7 (Table 1), besides similar conditions for ESP, which ranges from 22 to 38%. These attributes associated with subsuperficial clay accumulation identify this profile as Abruptic Solonetz [34].

On a thin section from Btn1 horizon, micromorphological description indicates a single spaced porphyric c/f related distribution pattern [31], with abundant channels and cavities (Table 3). Iron hypocoatings were typic, commonly associated with the cavities and channels formed by pedological processes [30]. On the Btnk thin section, the c/f related distribution is also porphyric, with Fe hypocoating and carbonate nodules. The smaller carbonate nodules are clustered, with creamy color interference and irregular shape (Fig 6B). Some impure reddish clay infilling is associated with carbonate precipitation in overlap, or even as incomplete infilling of the nodule nucleus (Fig 6C and Fig 6D). Small nodules arranged in circular form can also be observed at different stages of dissolution (Fig 6E and Fig 6F). The nodules were detached from the soil matrix and dated by AMS at approximately 660 years BP (Table 2).

At around 200cm in depth, a laminar clayey layer (C-horizon) with no evidence of pedogenic processes was observed. In order to understand parental material, an undisturbed sample was collected from C horizon for thin section confection. Its related distribution is massive, with fissural pores in horizontal orientation [31]. Several pedofeatures were observed, such as Fe/Mn depletion, hypo-coating and quasi-coating, all of which were associated with smaller fissures. Carbonate was also observed as a lens with a thickness of ~1000μm (Fig 6G). At a magnification of 1000 times, diatom structures were observed embedded in the carbonate lens (Fig 6H). This layer was followed in depth by a blanched sandy layer, dated by OSL at 54,200 years BP (Table 2), representing when the material received light for the last time.

#### Site 3

Soil morphology shows a 15cm thick E horizon. The underlying horizon has 3.5 times more clay content, pH values of 9.1 and an ESP of 34% (Table 1), it is also lighter in color, with abrupt transition between the E and Btn horizons (Table 2). Besides the high ESP value, EC is less than 0.5mS m^-1.^ The underlying horizon is reddish, shows powdered carbonate associated with fissures and old root channels, with a pH value of 8.8, an ESP of 35% and low EC, identifying this as a Btnk horizon. Beneath 130cm and underlying Btnk, the soil matrix is sandy with great variation in color. Despite decreases in pH and EC values in C horizons, ESP remains higher than 30%. This soil was identified as Abruptic Stagnic Solonetz [34].

The Btnk thin section presents fractured carbonate associated with voids among peds (channels), with creamy color interference (Fig 7B). Quartz grains embedded in carbonate show dissolution features, where both grain parts have optical continuity and serrated grain borders [37] (Fig 7C and 7D). The Btnk coarse fraction contains a small quantity of alterable primary minerals, such as biotite, feldspars and plagioclases (Table 3, Fig 7A). Iron depletion features occur associated with channels (Fig 7E and Fig 7F).

#### Site 4

The E horizon has a thickness of 20 cm, which is relatively small compared to the other sites. The underlying horizon is the thickest Btn horizon of all study sites, with 130cm thickness and yellowish color (Fig 5D). Clay content constantly increases with depth, starting with 50g kg^-1^ in the A and E horizons, reaching 450g kg^-1^ in the Cg horizon (Table 1). ESP was the highest of all the sites, varying from 51 to 60 in Btn2 and Btn1 horizon, respectively. Besides high ESP and pH values, EC values were no more than 0.71 dS m^-1^. This soil was identified as Abruptic Stagnic Solonetz [34].

**Fig 8:**
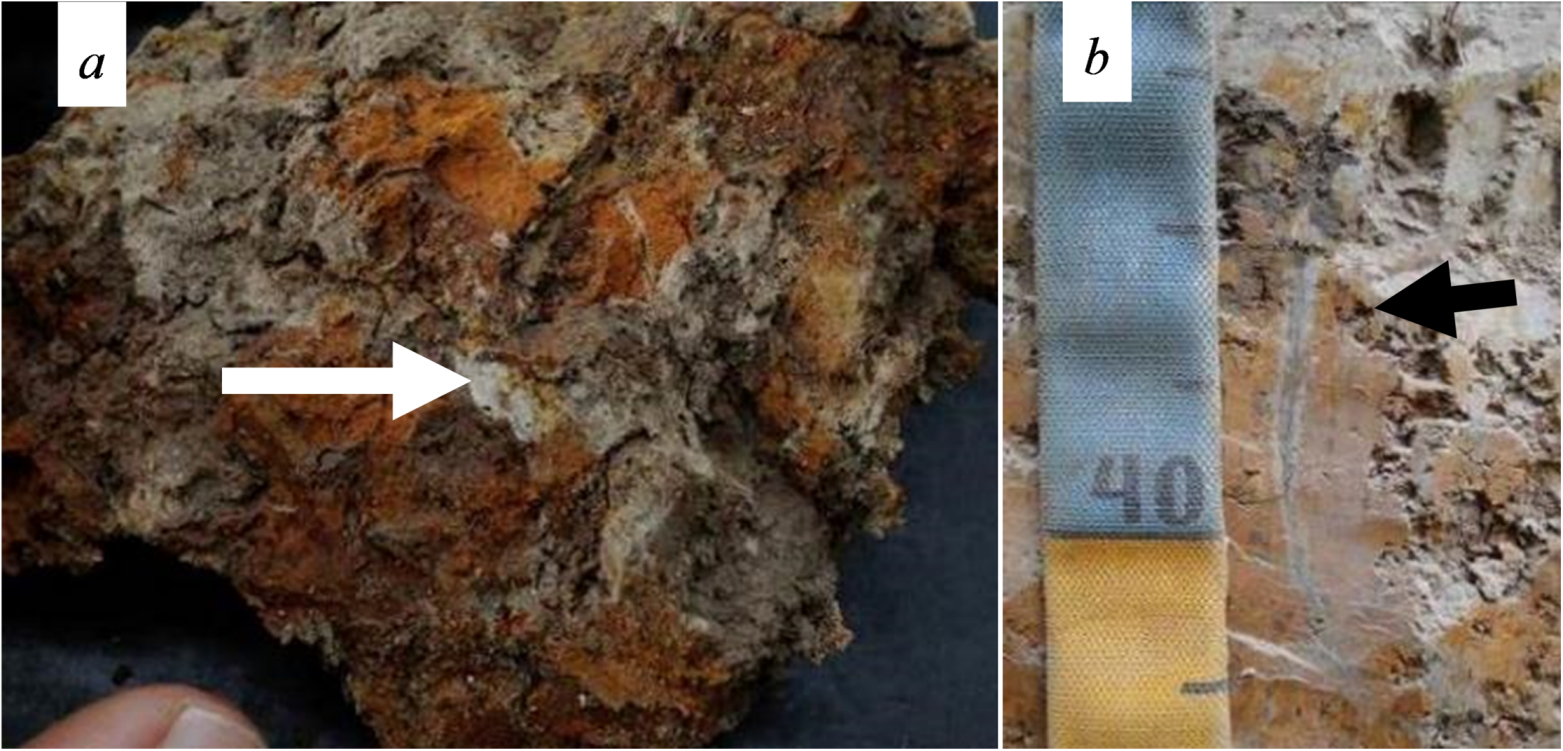
Morphological features from site 4. (A) Powdered white material. (B) Fe depletion in root channels, an incipient stage of ferrolysis process.

Some whitened features were observed in old biological pores (Fig 8A), which do not present effervescence when in contact with HCl, refuting carbonate presence. Redox features associated with root channels can be observed at the top of Btn1 (Fig 8B). Undisturbed sampling was performed in this part, in order to produce a thin section.

Btn1 presents a massive microstructure [30–31] and coarse material composed exclusively of quartz (Table 3). Only clay coating, associated with channels, was observed on the thin section, with yellow-red color, without lamination. Related distribution pattern is close porphyric, porosity is quite low and made up of fissures and cavities.

#### Site 5

The albic horizon (E) has a thickness of 80cm, with 800g kg^-1^ of sand, and ESP of 33% (Table 1). The underlying horizon (Btgn) is lighter in color, presenting twice the clay content of E horizon, has a columnar structure, broken and abrupt E/Btgn transition and an ESP of 49. The E horizon forms tongues into the natric horizon (*albeluvic glossae*) and temporal saturation with surface water (stagnic properties) is evidenced by Fe and Mn nodules, at a frequency of <5% in volume, with strong effervescence in contact with H_2_O_2_ [34]. The soil from site 5A was identified as Abruptic Stagnic Solonetz [36].

**Fig 9:**
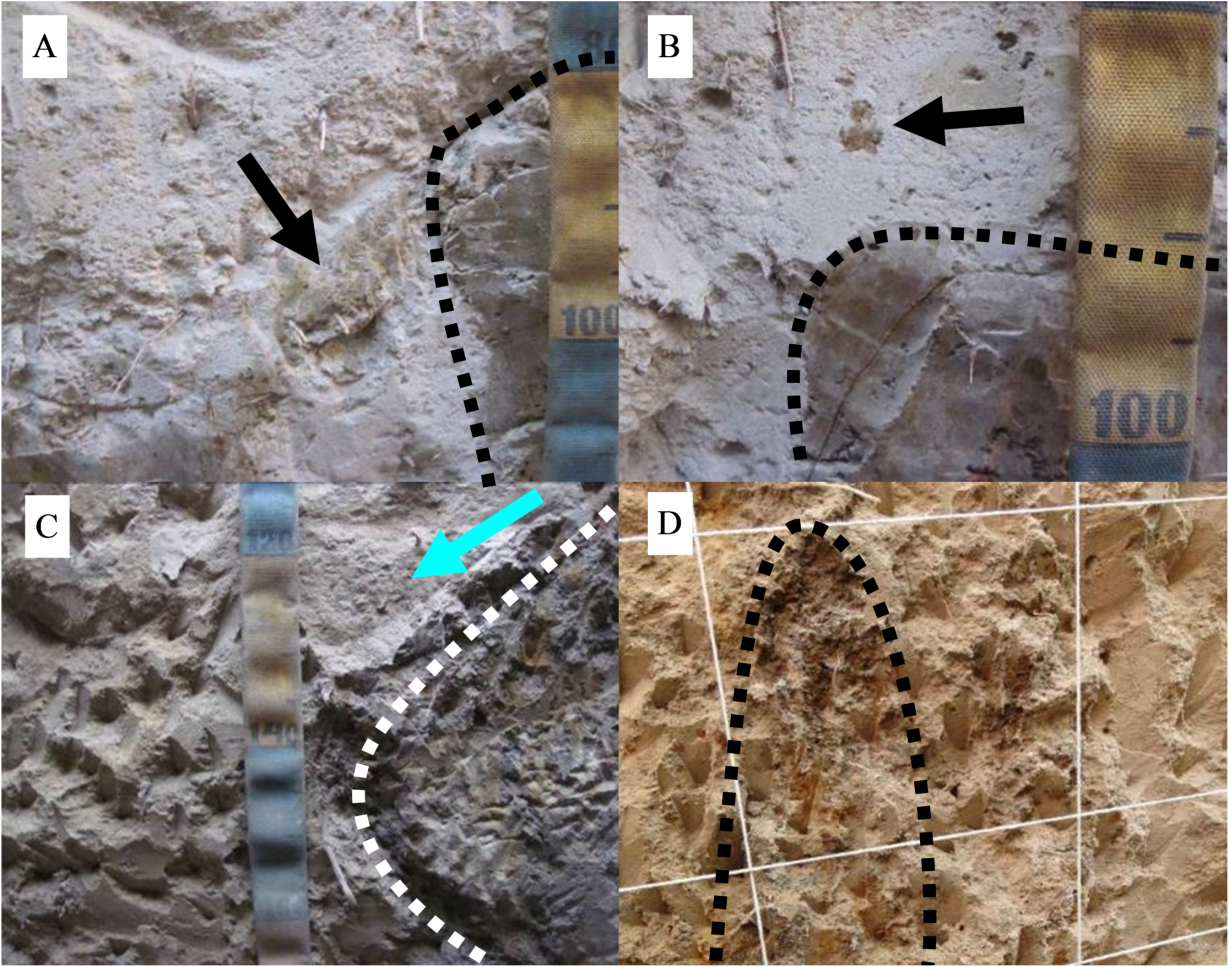
Bt degradation features from site 5. (A) Remnants of Btgn horizon inside the E horizon in trench5B. (B) Broken transition. (C) Arrow indicates the preferred direction of the lateral flow of water at the abrupt transition between E and Btgn. (D) Feature that looks like a remnant of the columnar structure observed in trench5A, in the same sector.

Remnants of the Btgn were observed in the midst of the E horizon (Fig 9A and Fig 9B). Several boreholes were made with the aim of establishing the extension and topography of the upper limit of Btgn (Fig 4A and 4B), which presents as basin like with an extension matching the mound. For the thin section an undisturbed sample from the E/Btgn transition was collected.

**Fig 10:**
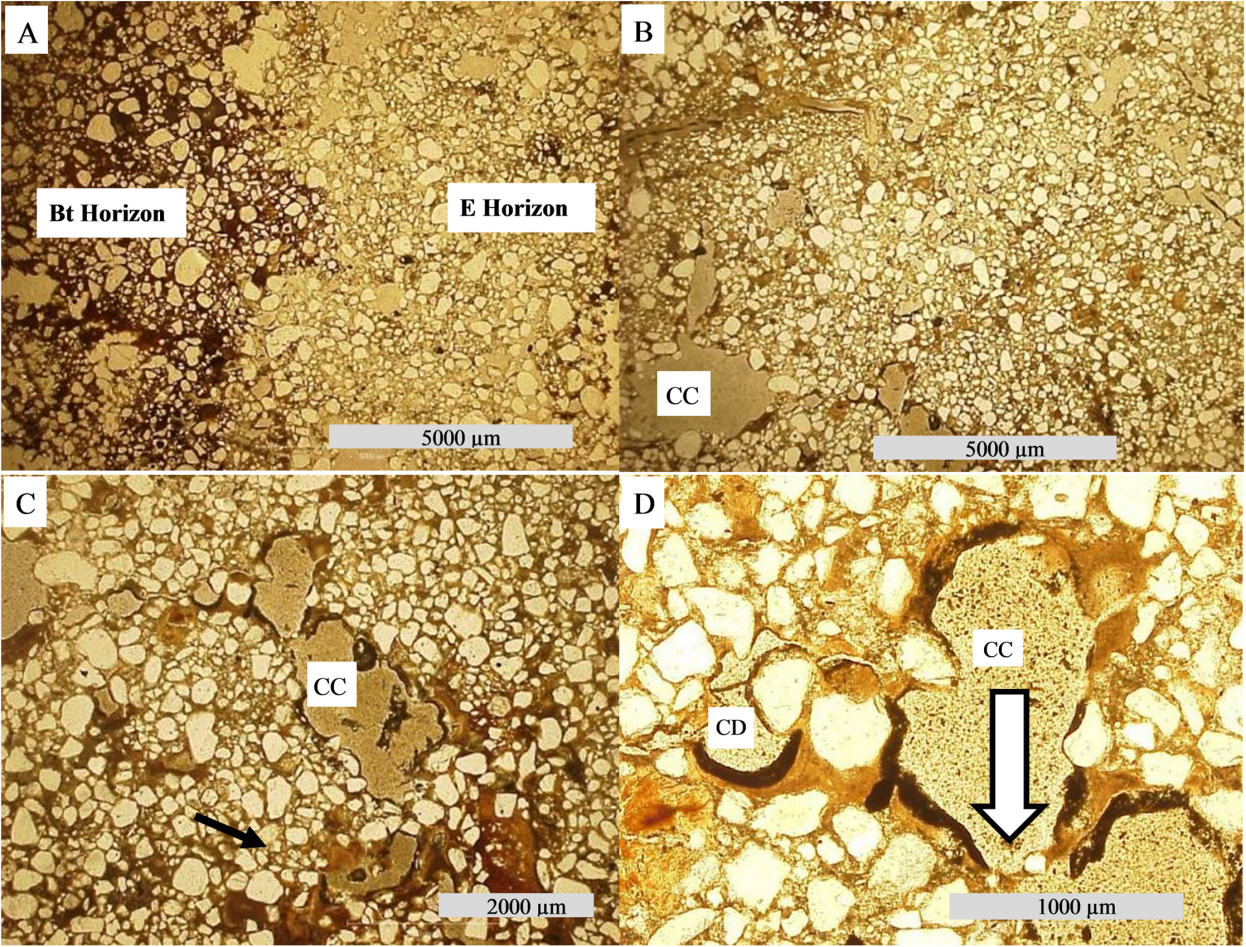
Photomicrographs from soil thin sections. (A) E and Btgn horizons from site 5 **–**abrupt contact with a significant presence of cavities (PPL). (B) Features of clay eluviation and cavity coalescence–CC. (C) Mamelonar void walls. (D) Features of Fe and clay coating in a dissolution void–CD, and cavity coalescence–CC. At the center of the photomicrograph the arrow indicates a thin layer of clay, which tends to be dissolute, forming cavity coalesce.

In the micromorphological description we observed that quartz grains from E and Btgn horizons are of similar size and shape (Fig 10A). For the micromorphological description, we indicate two zones on the thin section (Table 3, Fig 10A), one corresponding to En (Zone 1), and another corresponding to Btgn (Zone 2). Vugh porosity is frequently observed in Zone 2, at times with coalescence of two or more vughs [31]. In the void wall, impure brownish clay infilling is frequent, juxtaposed by anisotropic material infilling, probably Fe/Mn, however neither coating presents lamination features (Fig 10C and Fig 10D).

In profile 5B, broken and abrupt lateral transition can be observed from the Btgn/E horizon (Fig 9C). Small columnar structures turned towards the floodplain were observed, which are likely a remnant of larger columnar structures present in profile 5A (Fig 9D). Underlying the Btgn there is a more clayey grayish layer, which is identified as Cg, with flat topography, presenting high humidity, even in the driest period.

## Discussion

### Past processes

Some features observed in mound soils are not compatible with the current hydropedological conditions or biological activity, and we therefore associated them with past processes and climates. An example of these features is high ESP and pH values that commonly occur in semiarid regions and soils with quite low drainage. Despite not being currently observed, there is strong evidence that in the past there were lakes in the study area.

**Fig 11:**
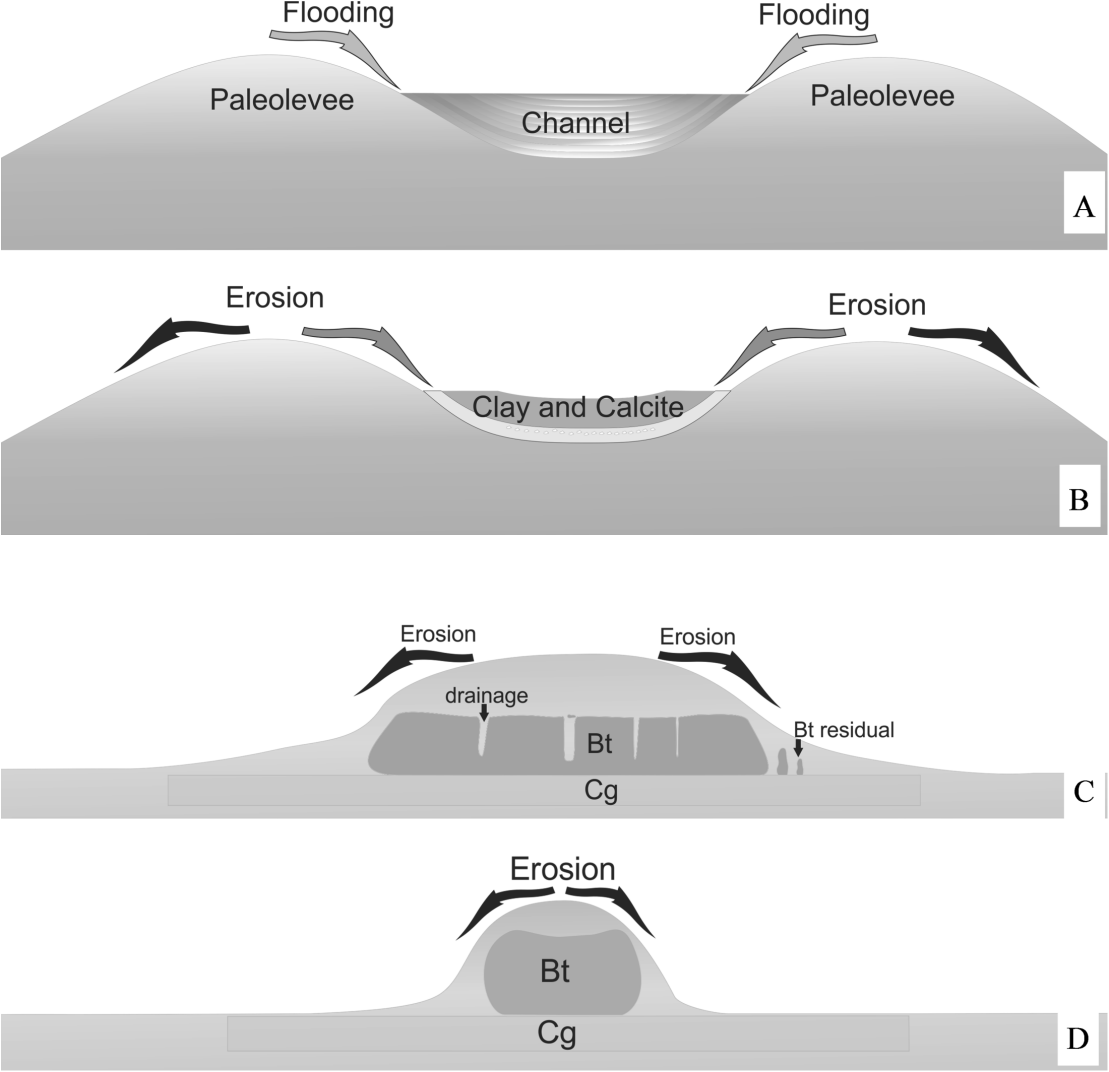
Model for mound, carbonate and natric character formation. (A) Flooding process raising higher parts of the landscape. (B) Erosive processes acting on the mound, with thick sediment burying clay and carbonates which becomes a subsurface layer. (C) Narrowing of the structure through erosion. (D) Thickening of the E horizon and formation of preferential drainage paths and remnants of the Bt horizon. At the most advanced stage of alteration and erosion, mound fragments will be narrowed.

Morphological features corroborate this hypothesis, for example the depression observed at site 1, is probably related to a former river channel enclosed by fluvial avulsion, which created a lake on the upper part of the landscape (oxbow lake). The laminar clayey structures at Site 2 (Fig 6G) also suggest that an old river channel was abandoned, becoming a low energy environment, transporting lighter sediments such as clay and silt. Sediments deposited under such conditions are finer than surrounding areas, hampering water percolation, thus, floodwater was subject to evapotranspiration (Fig 11A and Fig 11B). The huge diatomaceous presence in the carbonate lens (Fig 6H) corroborates the hypothesis that this material was deposited/formed in lacustrine environments [37–38].

Over several flooding cycles, the lake water became more and more concentrated with carbonate and clay minerals precipitating, once the solubility constant was reached [39–40]. The precipitation of clay minerals and carbonates elevates the saturation of Na^+^ in the soil exchange complex, resulting in a major pathway for sodification (ESP ≥ 15%) and natric horizon formation in mound soils [24,41]. Concomitant to Na^+^, other basic cations were concentrated, elevating the pH value. The Bt thin section from site 3 shows quartz fragments embedded in carbonate, with serrated border and optical continuity, suggesting that these nodules were formed a long time ago (Fig 6D), long enough for the alkaline pH to have dissolved the quartz grain [42–43].

The OSL dating indicates that the most recent material was deposited in the mound from 21,500 years BP, thus the events described before are compatible with climatic variations observed by McGlue *et al*. [44]. According to these authors, between 11,000 and 5,300 years BP, lakes on the Pantanal sedimentary basin stood at low levels, favoring ion input and concentration. From 5,300 to 2,600 years BP, the region experienced a period of severe drought, with no water input into lakes, promoting strong ion concentration.

During the last 2,600 years BP, the climate of the study site has become more humid and floods have become stronger and more recurrent, increasing erodibility [23,44]. Thus, the more elevated features have undergone new processes, in which the ridges of the oxbow lakes (paleolevees) have been eroded and the old lacustrine deposits covered. Therefore, higher parts of the landscape resulted from erosive processes, and remain on the Pantanal as a result of support from the soil horizon with clay accumulation.

On more preserved mounds, for example at sites 1 and 2, features inherited from depositional processes are still evident, refuting the theory of construction through biological activity. Clear or abrupt transitions between A and B-horizons, concomitant to sand of different sizes/shapes between horizons, provides evidence of sedimentary processes [14]. Thus, we can assume that higher parts of the landscape are inherited from paleolevees. In some cases, paleolevees increased their width through lateral migration of river courses. This last process is the result of natural silting of the river channel, resulting in energy loss. As a result, the watercourse tends to follow a pathway of lower energy which, in flood events, may result in crevasse spray and striking textural differences [45].

### Current processes

The current climate imposes new hydropedological processes in the Pantanal, resulting in landscape modeling. The more humid climate inhibits pedogenic carbonate precipitation from flooding in the Northern Pantanal. With the exception of the calcite lens at the trench bottom in Site 2, other carbonate features show signs of dissolution/remobilization, mainly in pore walls. These precipitations occur as coatings, infillings, nodule clusters (without nucleus) or nodules distributed in a circular shape (Fig 6E and Fig 6F). This last arrangement suggests that they belonged to a larger nodule, but were dissolved during the wetter climate of the last 2,600 years BP [44], and constitute a record of the driest preterite environments [46]. The short rainfall season is insufficient for complete removal and during the dry period the water in soil voids evaporates quickly, so carbonates precipitate again. Precipitations associated with channels are subject to remobilization through CO_2_ changes as a result of direct rainfall [47].

Concomitant to carbonate dissolution and partial elimination, other soluble salts tend to be leached, so ESP becomes greater and greater. High ESP is a major factor in argilluviation, which can be observed through coating and infilling pedofeatures, as observed on the thin sections [30,48]. When soil is made wetter through direct precipitation, colloids disperse and migrate in depth, concomitant with water and basic cations [49].

Argilluviation was observed in soils from more preserved mounds to more eroded mounds, suggesting that even at incipient stages of pedogenesis, it is one of the principal processes in the hierarchy. This process is essential to E horizon formation, which has a sandy texture and is more susceptible to erosion processes. Chronologically, argilluviation is a current process, evidenced by impure reddish clay coatings in the middle of carbonate nodules (Fig 6C and 6D). The lamination of clay coatings, which indicates several events of clay deposition [48], may be inhibited in sodic soil [50].

In the most eroded areas, agilluviation is at a more advanced stage, infilling the functional channels, limiting soil drainage and promoting hydric episaturation. Initially, this process results in Fe oxide reduction (Fe^3+^ to Fe^2+^) and depletion at the extremities of aggregates (Fig 6F) [51]. Alternation between hydric saturation and drought become more common, as do changes in iron between Fe^3+^ and Fe^2+^. This process results in occasional concentrations of H^+^, destabilizing the clay minerals and promoting their hydrolysis, which is also known as ferrolysis [49,52]. However, the Fe depletion has a secondary role, evidenced in some horizons (Fig 7F). Reddish clay coatings indicate that Fe oxides (hematite) may migrate in depth (Fig 6A), without the reduction of Fe^3+^.

The chemical analysis indicated the soil at Site 5 as the most acidic (Table 1), which is likely due to cation leaching and ferrolysis. The dissolution voids observed on the thin sections from the upper part of the Bt horizon and in the abrupt transition between E/Bt horizons, provide clear evidence of this process. This situation is the opposite of that observed by Van Ranst and De Coninck [53], which associated contrasts in soil texture to argilluviation. With the advance of the ferrolysis, coalescence of the voids can also be observed (Fig 10C and Fig 10D), resulting in voids with mamelonar wall arrangements with Fe coatings, which is clear evidence of temporary hydromorphism at this position of the soil profile [54].

Both ferrolysis and argilluviation are responsible for sandy matrix formation and thickening of the E horizon. The presence of fragments from the Bt horizon in the midst of the E horizon is evidence of its formation through Bt degradation [55]. Once the matrix becomes sandy, its resistance to erosion is drastically reduced. When this material stands at the edges of the paleolevees and is subject to flood waters, it is eroded and results in mound size reduction, similar to that observed in mounds associated with biological activity [1].

**Fig 12:**
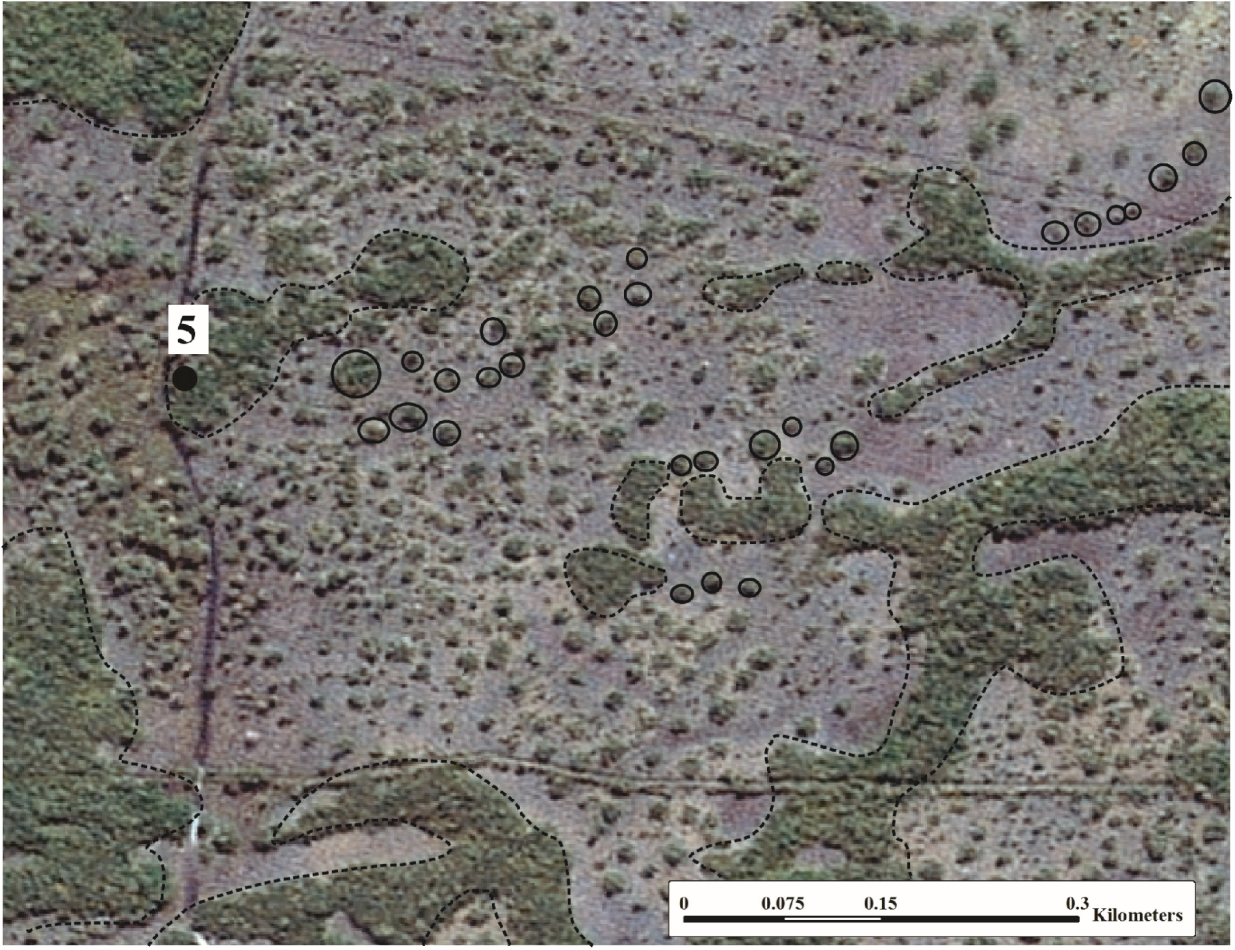
Satellite image showing mound alignment. Largest paleolevees (dotted lines) and some aligned mounds (continuous circles). Printed under a CC BY license, with permission from “Secretaria de Estado de Planejamento do Mato Grosso” (SEPLAN-MT), original copyright 2008.

The mounds at the studied site are then segmented into small rounded structures, albeit preserving their original spatial distribution, aligned to the former drainage channel. It is possible to observe that the mounds of pedogenic origin are arranged like a “necklace of Rosary beads” (Fig 12), contrasting to that observed by Nascimento *et al*. [1]. These authors suggest that some of these features originate from biological activity, such as a strategy of shelter from flooding, and are randomly distributed on the flood plain.

It is probable that after mounds assume small dimensions, such as at site 5, they are then colonized by termites. These species homogenize the material, attenuating the migration of the colloidal fractions, forming more stable aggregates through the agglutination of the components. Such soil alteration can result in more stable structures that are consequently more resistant to erosion [1].

Thus, in revisiting the origin of the vegetated mounds of the Pantanal, we arrive at a “hybrid” model of formation [13], in which the processes are not excluding of one another, but complementary [5].

## Conclusions

The origin of mounds in the Pantanal is a result of multiple successive processes. Their more elevated position on the landscape is inherited from paleolevees, formed through constant changes of river courses. High ESP values and calcium carbonate precipitations were produced in past drier climates and lacustrine environments.

The current, more humid, climate promotes degradation of soils from paleolevees. The waters from direct precipitation, associated with soils of sodic character, promote argilluviation and subsequent ferrolysis. Both ferrolysis and argilluviation result in E horizon thickening and Bt degradation. The sandy E horizon favors erosion of the paleolevees by the waters from flood events. At the final stage of transformation, floodplains coalesce and promote faster drainage of these areas.

The remnants of paleolevees then take on a circular format and may be colonized by termites, which in turn, homogenize the soil components and help preserve the more elevated parts against erosive processes.

## Supporting information

S1 Table Soil chemical, physical and morphological analysis. (XLSX)

S1 File KML. RPPN borders and sampled points.
(KML)
